# A Kinect-Based Segmentation of Touching-Pigs for Real-Time Monitoring

**DOI:** 10.3390/s18061746

**Published:** 2018-05-29

**Authors:** Miso Ju, Younchang Choi, Jihyun Seo, Jaewon Sa, Sungju Lee, Yongwha Chung, Daihee Park

**Affiliations:** Department of Computer Convergence Software, Korea University, Sejong City 30019, Korea; misoalth@korea.ac.kr (M.J.); ycc4477@korea.ac.kr (Y.C.); goyangi100@korea.ac.kr (J.S.); sjwon92@korea.ac.kr (J.S.); peacfeel@korea.ac.kr (S.L.); dhpark@korea.ac.kr (D.P.)

**Keywords:** agriculture IT, computer vision, depth information, touching-objects segmentation, convolutional neural network, YOLO

## Abstract

Segmenting touching-pigs in real-time is an important issue for surveillance cameras intended for the 24-h tracking of individual pigs. However, methods to do so have not yet been reported. We particularly focus on the segmentation of touching-pigs in a crowded pig room with low-contrast images obtained using a Kinect depth sensor. We reduce the execution time by combining object detection techniques based on a convolutional neural network (CNN) with image processing techniques instead of applying time-consuming operations, such as optimization-based segmentation. We first apply the fastest CNN-based object detection technique (i.e., You Only Look Once, YOLO) to solve the separation problem for touching-pigs. If the quality of the YOLO output is not satisfied, then we try to find the possible boundary line between the touching-pigs by analyzing the shape. Our experimental results show that this method is effective to separate touching-pigs in terms of both accuracy (i.e., 91.96%) and execution time (i.e., real-time execution), even with low-contrast images obtained using a Kinect depth sensor.

## 1. Introduction

When caring for group-housed livestock, the early detection and management of problems related to health and welfare is important. In particular, the care of individual animals is necessary to minimize the potential damage caused by infectious disease or other health and welfare problems. However, it is almost impossible for a small number of farm workers to care for individual animals on a large livestock farm. 

Several studies have recently used surveillance techniques to automatically monitor livestock [1,2,3,4]. In this study, we focus on video-based pig monitoring applications with non-attached (i.e., non-invasive) sensors [5,6,7,8,9,10,11,12,13,14,15,16]. Furthermore, we employ a top-view depth sensor [17,18,19,20,21,22] due to the practical difficulties presented in commercial farms where the light is turned off at night (i.e., light fluctuations, shadowing, cluttered backgrounds, varying floor status caused by urine/manure, etc.). In fact, we previously reported results for Kinect-based pig detection [22]. The 24-h tracking of individual pigs requires for touching-pigs in a crowded room to be separated. However, the depth values obtained from low-cost sensors, such as Microsoft Kinect, may be inaccurate, and the input video stream data needs to be processed in real-time through an online analysis. 

In this study, we propose a low-cost and real-time method to separate touching-pigs. In particular, caring for weaning pigs (25 days old) is the most important issue for pig management due to their weak immunity. Therefore, we aim to develop a method to separate weaning pigs, and the contributions of the proposed method can be summarized as follows:

- Touching-pigs are separated using a low-cost depth camera, such as Microsoft Kinect. However, the size of an 8-kg weaning pig is much smaller than that of a 100-kg adult pig. Furthermore, if we install a Kinect at 3.8 m above the floor to cover a larger area (i.e., minimizing the installation cost for a large-scale farm), then the accuracy of the depth data measured from the Kinect sensor is significantly degraded. To handle the low-contrast images, we consider both deep learning-based object detection techniques and image processing techniques.
- A real-time solution is proposed. Separating touching-pigs is a basic low-level vision task for intermediate-level vision tasks, such as tracking, and/or high-level vision tasks, such as aggressive analysis. To complete all of the vision tasks in real-time, we need to decrease the computational workload of the separation task. We apply the fastest deep learning-based object detection technique (i.e., You Only Look Once, or YOLO [23]), and develop lightweight algorithms to evaluate the quality of the YOLO output and find the possible boundary line between the touching-pigs by analyzing their shape.

In the following, we use the terms *segmentation* and *separation* interchangeably, and the remainder of this paper is structured as follows. Section 2 summarizes previous methods for segmentation. Section 3 describes the proposed method to separate touching-pigs in various touching cases (i.e., both “easy-to-separate” and “difficult-to-separate” cases). The experimental results are presented in Section 4, and the conclusions are presented in Section 5.

## 2. Background 

**Figure 1:**
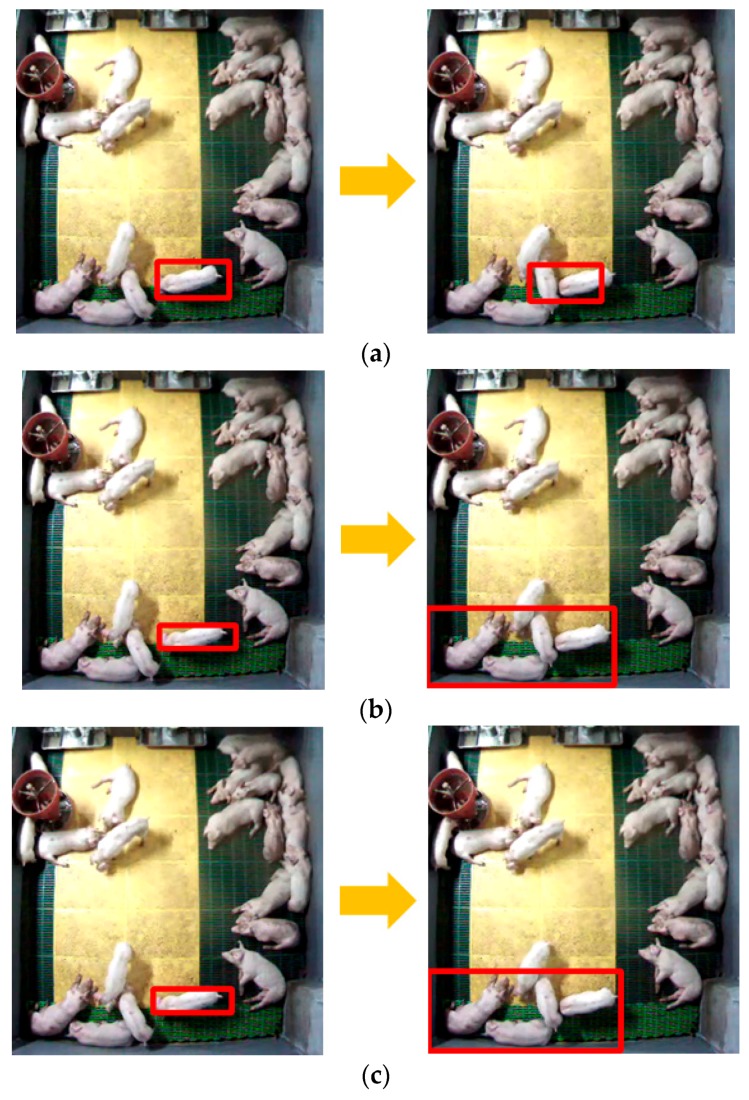
Tracking failures caused by touching-pigs: (**a**) results of the mean shift algorithm [24]; (**b**) results of CAM shift algorithm [25]; (**c**) results of Kalman filter algorithm [26].

The present study contributes to our final goal of 24-h automatic pig behavior analysis by focusing on identifying individual pigs based on pig segmentation. Previous studies performed segmenting and tracking [15,16], but the mean times between tracking failures were less than a few minutes. For example, Figure 1 shows tracking failures with the simplest tracking algorithms (i.e., mean shift [24], continuously adaptive mean (CAM) shift [25], and Kalman filter [26]) implemented using the open-source software OpenCV [27]. Clearly, typical tracking algorithms can segment and track each isolated moving pig correctly. When multiple moving pigs are very close to each other (we designate these adjacent “moving” pigs as **touching-pigs**), the tracking algorithms cannot identify each pig, and thus tracking failures occur. Furthermore, the higher the pig room density, the more difficult it will be to segment the pigs in the room [14]. Therefore, the key problem when segmenting and tracking weaning pigs continuously during the automatic analysis of pig behavior is to separate touching-pigs in a crowded environment.

As explained in Section 1, we consider a low-cost depth camera such as Microsoft Kinect because of the practical difficulties (i.e., light fluctuation, shadowing, cluttered background, varying floor status caused by urine/manure, etc.) and 24-h monitoring (i.e., even with no-light conditions at night). However, a low-cost Kinect camera has a limited distance range (i.e., up to 4.5 m), and the accuracy of the depth data measured by a Kinect decreases quadratically as the distance increases [28]. Thus, the accuracy of the depth data of the Kinect is significantly degraded when the distance between it and a pig is larger than 3.8 m. Considering this difficulty, it is very challenging to separate 8-kg weaning pigs using a Kinect camera installed 3.8 m above the floor.

**Figure 2:**
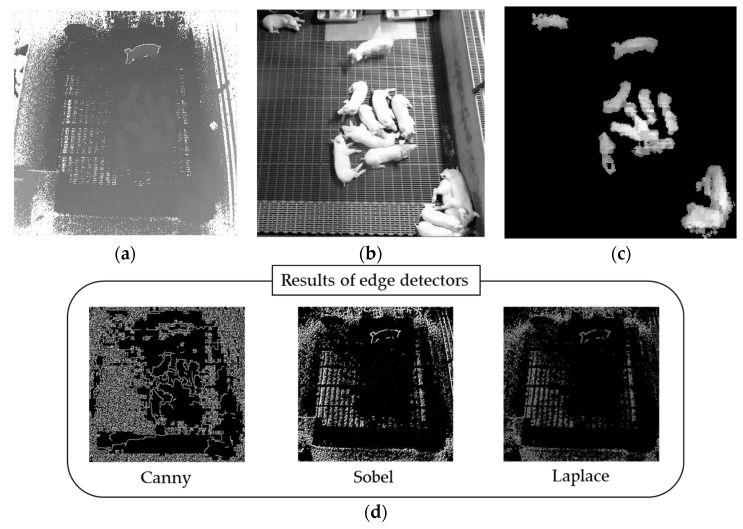
Difficulty of separating touching-pigs: (**a**) depth image; (**b**) gray image converted from a corresponding color image (during daytime); (**c**) result of contrast limited adaptive histogram equalization (CLAHE) [29] after background subtraction; (**d**) results of the edge detectors [30].

For example, the images in Figure 2a,b respectively show a Kinect depth image and a gray image converted from the corresponding color image. We can clearly see the difference in contrast between the two gray images. In order to improve the low-contrast image shown in Figure 2a, we applied one of the most widely used techniques (i.e., contrast limited adaptive histogram equalization, CLAHE [29]) to improve the low-contrast images, such as for computed tomography (CT)/magnetic resonance imaging (MRI) in biomedical applications. Figure 2c shows the result of CLAHE after background subtraction, and it is difficult to find the possible boundary lines between the touching-pigs. As shown in Figure 2d, various edge detectors [30] could not detect the possible boundary lines either.

Table 1 summarizes some of previous methods that have been used for segmentation. Some of the methods solved the separation problem of touching-objects (i.e., “management of touching objects = Yes”, as shown in Table 1), whereas some others did not (i.e., “management of touching objects = No”, as shown in Table 1). To analyze low-contrast images, such as CT/MRI and thermal/depth, many previous methods employed time-consuming optimization techniques, such as active contour (snake), level set, graph cut, etc. Although online monitoring applications should satisfy the real-time requirements, many previous results did not specify the processing speed or could not satisfy the real-time requirements. To the best of our knowledge, this is the first report on separating touching objects in real-time with low-contrast images obtained using a Kinect sensor. By carefully balancing the trade-offs between the computational workload and accuracy, we propose a lightweight method with an acceptable accuracy with the final goal of achieving a “complete” real-time vision system, consisting of intermediate and high-level vision tasks, in addition to low-level vision tasks.

## 3. Proposed Approach

In this study, we assume that the size of each pig is similar, and that each moving pig is detected using the Gaussian mixture model (GMM). By analyzing the size of each connected component of the GMM result, we can recognize each connected component as a single pig or a group of pigs, and we try to separate the two adjacent moving pigs (i.e., touching-pigs) for high-level vision tasks, such as with an aggressive analysis. It is well-known that each pig sleeps most of the time, and the moving activity of each pig (measured with GMM) was observed with a probability of less than 1% on the average [60]. The case of touching-pigs with more than three moving pigs is very rare, and thus, we focus on the case of touching-pigs with two moving pigs in this study. Algorithm 1 displays the overall procedure of the proposed method. The details of the preprocessing steps for the depth images (e.g., noise removal and background subtraction) can be found in Kim et al*.* [22].

**Figure 3:**
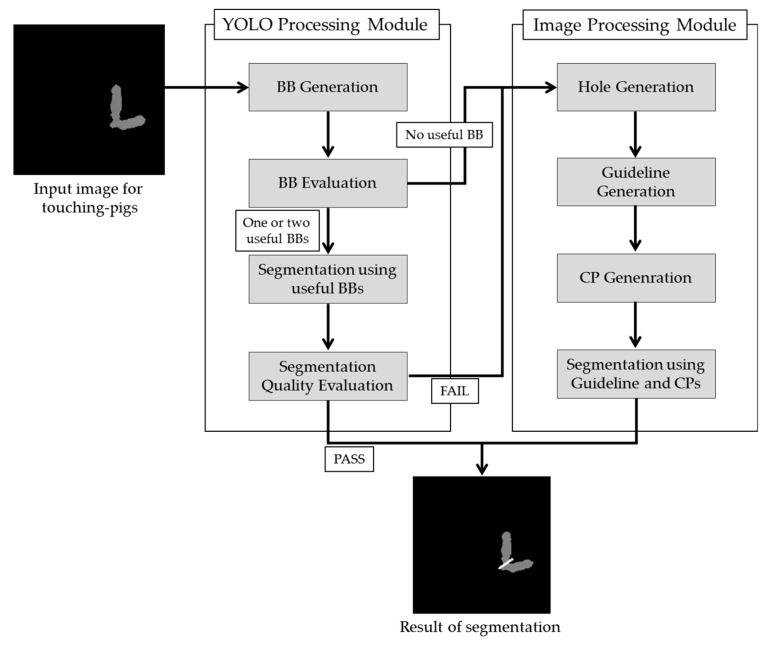
Overview of the proposed method.

Our segmentation method consists of two modules (YOLO Processing Module and Image Processing Module), and Figure 3 presents an overview of our method for touching-pigs detected using the Kinect depth camera. Although YOLO can detect a single pig with acceptable accuracy, YOLO may not separate touching-pigs with acceptable accuracy. Therefore, we first apply YOLO to the touching pig to obtain the bounding boxes (BBs) from the YOLO Processing Module. One or two useful BBs are selected by checking the quality of each BB, and then the touching-pigs are segmented with the useful BBs. If the quality of either the BBs or the segmentation is not satisfied, then we try to find the possible boundary line between the touching-pigs by analyzing the shape. Since any hole in the touching-pigs can be useful to find the possible boundary line, we first check for any hole in the Image Processing Module. If the touching-pigs do not have such a hole, then we artificially generate hole(s) by analyzing the outline of the touching-pigs. Then, we generate both a guideline (i.e., part of the boundary line) from the hole information and concave points (CPs) from the outline information. Finally, we conduct segmentation using the guideline and the CPs.

**Algorithm 1** Overall algorithm with the proposed methodInput: An image sequence from a depth information video Output: An image sequence where touching-pigs are individually separated *// Load a depth information video* Seq=Load(depth_video) *// Remove noises in depth images* $Seq_{Interpolate}=\mathrm{SpatioTemporalInterpolate}\left(Seq\right)$ *// Perform background subtraction* $Seq_{BS}=\mathrm{BackgroundSubtract}\left(Seq_{Interpolate}\right)$ *// Detect moving pigs only* $Seq_{GMM}=\mathrm{GMM}\left(Seq_{BS}\right)$ *// Separate touching-pigs using the proposed method* $\mathrm{for}\;\left(i=1;\;i\;\leq \mathrm{the}\;\mathrm{number}\;\mathrm{of}\;\mathrm{connected}\;\mathrm{components}\;\mathrm{in}\;Seq_{GMM};\;i++\right)\;$  If size of each connected component $I_{in}^{i}\left(Seq_{GMM}\right)$
>
*size of a single pig*
    Go to YOLO Processing Module with $I_{in}^{i}\left(Seq_{GMM}\right)$
    Save the separated results of the touching-pigs Return;

### 3.1. YOLO Processing Module

• *BB Generation*

YOLO is a convolutional neural network (CNN)-based object detection technique, and it uses the grid method, which allows efficient object detection in real-time. In particular, this study uses the YOLO9000 technique. YOLO9000 offers improved performance compared with YOLO by applying batch normalization on all of the convolutional layers and multi-scale learning on the training data. The input image contains only touching-pigs, as we assumed earlier, and it is applied to YOLO. Then, we can obtain the image with bounding boxes (BBs) for the possible boundary of each pig in touching-pigs. Note that we assume YOLO was trained with images with ground truth-based BBs for the individual pig of each touching pig. 

In YOLO, the input image is equally and arbitrarily divided into a set of *S* × *S* grid cells, and *B* BBs are generated through each grid cell with x coordinate, y coordinate, width, height, and probabilities of existence of each object (i.e., a pig). In other words, through this step, each cell can generate a large number of BBs with object probability information that is higher than the threshold value (denoted as *BB_probability_th*). Also, YOLO is well known as one of the fastest techniques for object detection. In this study, we set *S*, *B*, and *BB_probability_th* as 13, 5, and 1, respectively.

• *BB Evaluation*

**Figure 4:**
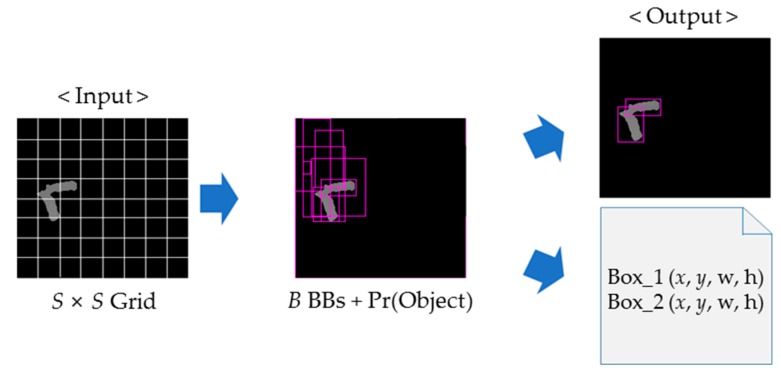
Selection of two useful bounding boxes (BBs) using YOLO.

**Figure 5:**
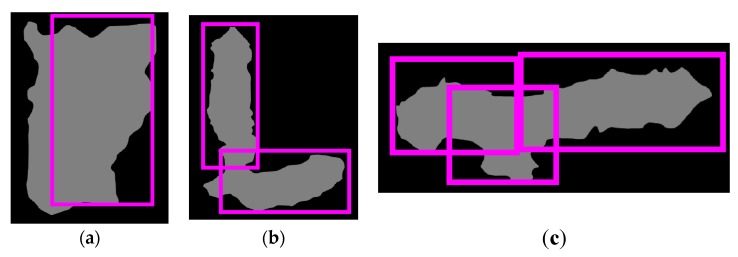
Three cases of YOLO results within the region of interest (RoI): (**a**) single BB; (**b**) two BBs; (**c**) three BBs.

After generating BBs, we need to select one or two useful BBs to separate each touching pig, and Figure 4 shows the selection results for two useful BBs. For BB evaluation, we first set the region of interest (RoI) as an enclosing box for each touching-pig, and we then remove the YOLO BBs that are not included in the RoI. As shown in Figure 5, YOLO can generate more than one BB within the RoI. 

To evaluate the usefulness of each BB within the RoI, we first compute the number of pig pixels within the RoI (denoted as *PP_RoI_*). Then, for each BB, we compute the number of pig pixels within the BB (for example, denoted as *PP_BB_A_* for “*BB_A*”) and check if 0.4 *PP_RoI_* ≤ *PP_BB_A_* ≤ 0.6 *PP_RoI_* (denoted as *BB size condition*).

Then, we compute the number of boundary pixels of the RoI (denoted as *BP_RoI_*). For each BB, we also compute the number of boundary pixels of the BB near the boundary pixels of the RoI (for example, denoted as *BP_BB_A_* for “*BB_A*”) and check if 0.2 *BP_RoI_* ≤ *BP_BB_A_* (denoted as *BB boundary condition*). If these two conditions are satisfied for a BB, then we refer to it as a *useful* BB.

Finally, if the number of useful BBs that passed from the previous step is zero, then we segment this case using the Image Processing Module (Section 3.2). On the contrary, if the number of useful BBs is two or more, then we need to compute the *BB confidence value* of each useful BB. Equation (1) shows the calculation of the BB confidence value for useful “*BB_A*”. That is, a higher BB confidence value means that the useful BB satisfies both the BB size and boundary conditions with a high confidence level.

(1) $$BB\;confidence\;value=\frac{BP_{BB\_A}-0.2BP_{RoI}}{\left|PP_{BB\_A}-0.5PP_{RoI}\right|}$$

**Figure 6:**
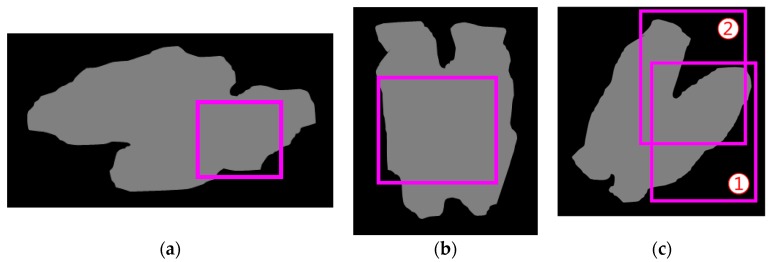
Illustration of the BB size, boundary, and coverage conditions: (**a**) the BB is not satisfied as useful according to the BB size condition; (**b**) the BB cannot be a useful BB, because it satisfies the BB size condition, but it cannot satisfy the BB boundary condition; (**c**) both BBs satisfy the BB size and boundary conditions, but the first BB ① can finally be a useful BB, because the second BB ② covers less than half of the remaining pig pixels (i.e., violation of the BB coverage condition).

Based on a descending order of the BB confidence value, we first select one useful BB with the highest BB confidence value (for example, “*BB_A*” and denoted as *first useful BB*). Then, we check whether each remaining useful BB covers at least half of the remaining pig pixels within the RoI (i.e., *PP_RoI_* − *PP_BB_A_*). If a remaining useful BB satisfies this condition (denoted as the *BB coverage condition*), then we select this useful BB (denoted as *second useful BB*) and pass these two useful BBs (i.e., first and second useful BBs) to the next step. Otherwise, we pass only one useful BB (i.e., first useful BB). In Figure 6, we illustrate the BB size, boundary, and coverage conditions. Note that the computational time for this step is negligible, because the number of BBs within the RoI is relatively small (i.e., typically, 1–3).

• *Segmentation Using Useful BBs*

**Figure 7:**
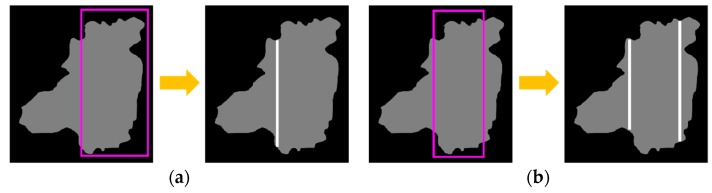
Segmentation with a single useful BB: (**a**) single boundary line; (**b**) two boundary lines.

If the number of useful BBs is one, and the number of boundary lines generated by the useful BB is one, then we segment this case with the useful BB (See Figure 7a). If the number of useful BBs is one, but the number of boundary lines generated by the useful BB is two, then we need to segment this case with the Image Processing Module (See Figure 7b). 

**Figure 8:**
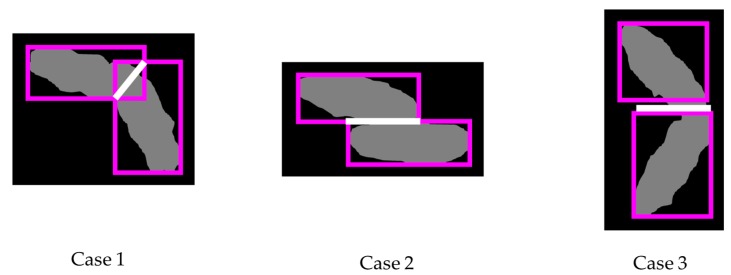
Segmentation with two useful BBs.

Finally, we need to segment the case of two useful BBs. Depending on the relative positions between two useful BBs, we classify this case into three cases (see Figure 8): Case 1 creates an overlapping rectangle, Case 2 creates an overlapping line, and Case 3 creates no overlapping rectangle or line. 

• *Segmentation Quality Evaluation*

**Figure 9:**
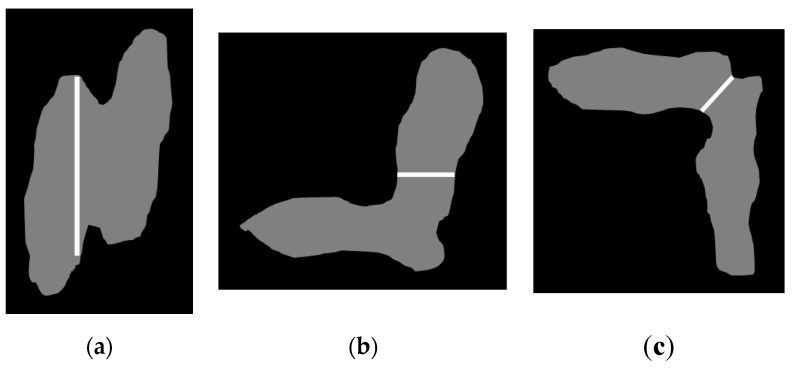
Illustration of the segmentation length and size conditions: (**a**) case of dissatisfaction for the segmentation length condition; (**b**) case of dissatisfaction for the segmentation size condition while satisfying the segmentation length condition; (**c**) case of satisfaction for both the conditions.

If a given case is segmented in the previous step, we need to determine the segmentation quality produced by the previous step. Based on our preliminary experiment, we found that the YOLO Processing Module produced an acceptable quality with short boundary line cases (i.e., the touching region between two pigs is relatively small), but an unacceptable quality with long boundary line cases (i.e., the touching region between two pigs is relatively large). Therefore, we first determine the length of the boundary line (denoted as *L_BL_*), and check if *L_BL_* ≤ *segmentation_length_th* (denoted as *segmentation length condition*). If this condition is satisfied, then we finally check the relative size of each separated pig, as determined by the boundary line. That is, we check if (the size of a larger pig/size of a smaller pig) ≤ *segmentation_size_th* (denoted as *segmentation size condition*). If these two conditions are not satisfied, then we segment this case with the Image Processing Module (Section 3.2). In this study, we set the *segmentation_length_th* as 20, because the length of a pig observed by our experiment is about 35 on average. We also set *segmentation_size_th* as 1.5. In Figure 9, we illustrate the segmentation length and size conditions, and the proposed method using YOLO BBs is summarized in Algorithm 2, which is given below. 

**Algorithm 2** Separation algorithm of YOLO Processing ModuleInput: An image of touching-pigs Output: An image of individually separated pigs *// BB generation* Generate BBs by YOLO *// BB evaluation* Set RoI Check BB size, boundary, and coverage conditions Determine useful BBs If the number of useful BBs is 0   Exit this function and go to Image Processing Module *// Segmentation using useful BBs*Connect (two points with one or two useful BBs) If the number of useful BBs is one and the number of generated boundary lines is two   Exit this function and go to Image Processing Module *// Segmentation quality evaluation* Check segmentation length and size conditions If conditions are not satisfied,   Exit this function and go to Image Processing Module Return;

### 3.2. Image Processing Module

• *Hole Generation*

**Figure 10:**
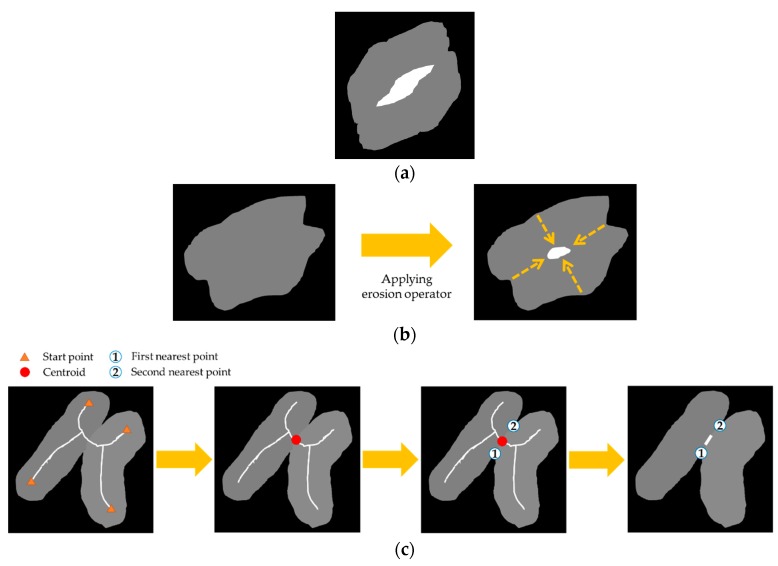
Illustration of natural and artificial holes: (**a**) natural hole; (**b**) artificial hole using erosion operator; (**c**) artificial hole using skeleton operator.

In the Image Processing Module, we separate the touching-pigs by analyzing the shape of the touching-pigs. Since any hole in the touching-pigs can be useful to find the possible boundary line, we first check for any hole within the touching-pigs. If the touching-pigs have such holes, then we use it (denoted as *natural* holes, for the purpose of explanation). Otherwise, we generate holes (denoted as *artificial* holes, for the purpose of explanation). The artificial holes can be simply generated by shrinking the outline of the touching-pigs. That is, we repeatedly apply a morphological erosion operator to the outline by *erosion_repeat_th* times (See Figure 10b). In this study, we set *erosion_repeat_th* as 8, because the width of a pig observed by our experiment is about 10 on average.

If a hole cannot be generated with the erosion operator, then we apply a skeleton operator (i.e., medial axis transform, MAT) to a reduced resolution image to reduce the time of the computation. After finding the skeleton, we apply skeleton tracing from the start points to find a centroid. We select the “first” nearest point on the touching-pigs outline from the centroid, and then select the “second” nearest point on the opposite outline from the centroid. With these two nearest points and the centroid, we can generate a hole of length 4 (See Figure 10c). The details of the skeleton tracing can be found in Chung et al*.* [61]. In Figure 10, we illustrate the natural and artificial holes.

• *Guideline Generation*

**Figure 11:**
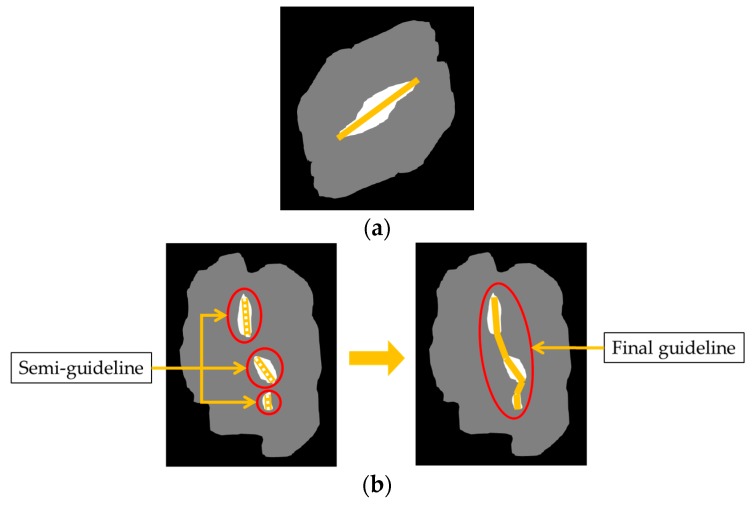
Generation of a guideline with a single hole and multiple holes: (**a**) Case 1: guideline with a single hole; (**b**) Case 2: guideline with multiple holes.

After the hole-generation step, we are able to take advantage of the hole information in any shape of the touching-pigs. In this step, we generate a guideline that can be a part of the boundary line by connecting the points in each hole. As shown in Figure 11, we first find two opposite points that are farthest from each other. Then, we draw a guideline by connecting the two points. If there are multiple holes (Case 2), we connect each guideline (denoted as a semi-guideline) of each hole in order to generate a final guideline.

• *CP Generation*

**Figure 12:**
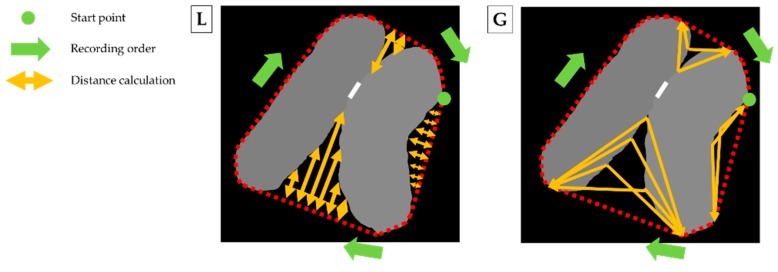
Illustration of two different types of time-series data L and G.

In order to find an acceptable boundary line, we additionally generate concave points (CPs) located on the outline of the touching-pigs. Based on our preliminary experiment, we found that at least one CP in the touching region was observed. However, the CPs can also be detected other than at the touching region, since a pig can bend its back (see Figure 12). It is very important to distinguish between the two cases. Therefore, we interpret two-dimensional outline data into one-dimensional time-series data, and utilize the time-series data as a tool to make a distinction between the two cases.

The CP generation step is as follows. We first apply a convex hull algorithm that was implemented using OpenCV, and we obtain a minimum polygon that contains an outline of the touching-pigs. The point segments of the polygon are stored in the list in counterclockwise order. The last stored segment is set as a start point of the time-series data. Two types of time-series data are generated by recording the distances from the minimum polygon and the outline of the touching-pigs clockwise. In a time-series data *L*, the distances of the vertical lines between the line segments of the polygon and the outline corresponding to each segment from the start point are recorded. Similarly, in time-series data *G*, the distances of each end point of the line segments and each point of the corresponding outlines from the start point are recorded. The generation process of the time-series data L and G are illustrated in Figure 12.

**Figure 13:**
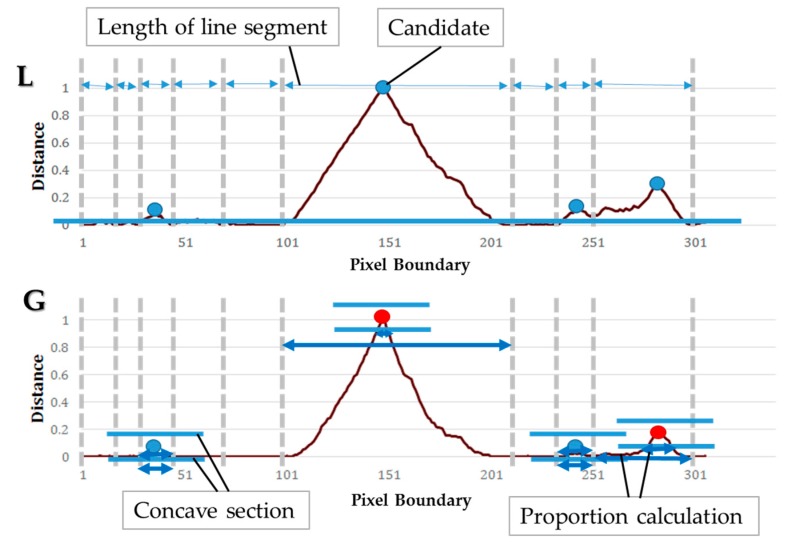
Generation of concave points (CPs) with two different types of time-series data.

One of the goals of this step is to determine whether the CPs are detected in the touching region or not. Figure 13 describes the generation of CPs with the two different types of time-series data L and G. The time-series data L represents the local curvature information, since it is globally irregular. On the other hand, the time-series data G represents the global curvature information, since it is locally irregular. Therefore, we extract maximum values corresponding to each line segment of the minimum polygon from the time-series data L, and set them as candidates of CPs (see blue points in Figure 13L). Then, we calculate a proportion, which represents a degree of concavity, by dividing a length of the concave section by the length of the corresponding line segment from the time-series data G (see arrows in Figure 13G). The interval of the concave section is set to each candidate value ± candidate value × 0.1 (see bars near the candidates in Figure 13G). More than two CPs cannot be created for the touching-pigs, and thus, if the number of the CPs is larger than two, we obtain only the two points with large distance values recorded in the time series data L as the final CPs.

• *Segmentation using Guideline and CPs*

**Figure 14:**
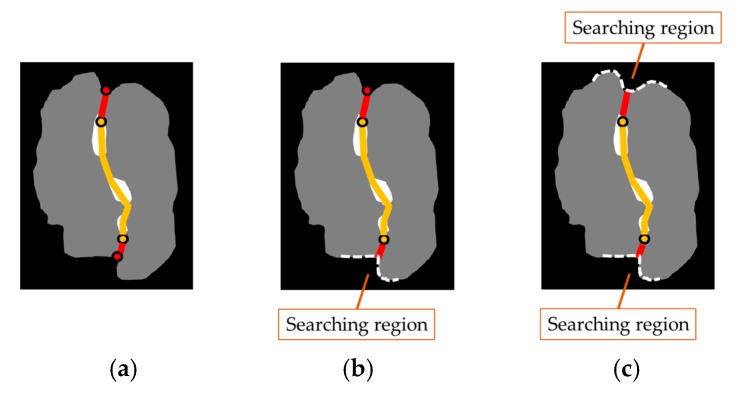
Segmentation with a guideline and CPs: (**a**) Case 1: two CPs; (**b**) Case 2: one CP; (**c**) Case 3: no CP.

The separation step is classified into three cases as shown in Figure 14. In Figure 14, the red points are the final CPs, and the yellow points are the end points of the guideline (yellow lines). First, if the number of the CPs is two (Case 1), then we just draw the possible boundary line by connecting the CPs and both end points of the guideline. Secondly, if there is only one CP (Case 2), then we connect the CP and an end point, which has a shorter distance from the CP between both end points of the guideline. Then, we set the opposite side of the CP to a searching region. We finally draw the possible boundary line by connecting the remaining end point of the guideline and the closest point in a searching region from the point. Finally, if there is no CP (Case 3), we select the closest point in the outline from any end points of the guideline. After connecting the guideline and the selected point, such as with Case 2, we set the search region. Finally, we connect the remaining end point of the guideline and the closest point located in the search region from the point. The proposed method using image processing techniques is summarized in Algorithm 3 and is given below.

**Algorithm 3** Separation algorithm of Image Processing ModuleInput: An image of touching-pigs Output: An image of individually separated pigs *// Hole generation* Check natural holes from the touching-pigs If any hole is not detected  Generate artificial holes with erosion or skeleton operator *// Guideline generation* Select two points with the longest distance in each hole Semi-guideline = Connect (two points of each hole) Guideline = Connect (all of the semi-guidelines) *// CP generation* ConvexHull (shape of the touching-pigs) Make time-series data L and G Find candidates of CPs with time-series data L Find at most two CPs from the candidates with time-series data G *// Segmentation using guideline and CPs* If the number of final CPs is two   Connect (guideline, concave points) Else if the number of final CPs is one   Connect (guideline, CP, selected point in the searching region) Else if there is no CP   Connect (guideline, the closest point, selected point in the searching region) Return;

## 4. Experimental Results

### 4.1. Experimental Environment and Dataset

In our experiment, the training for the object detection with YOLO was conducted in the following environment: Intel Core i7-7700K 4.20 GHz (Intel, Santa Clara, CA, USA), 32 GB RAM, Ubuntu 16.04.2 LTS (Canonical Ltd., London, UK), NVIDIA GeForce GTX1080 Ti 11 GB VRAM (NVIDIA, Santa Clara, CA, USA), and OpenCV 3.2. Furthermore, the test to separate the touching-pigs through the training model derived from YOLO was performed in the same environment.

**Figure 15:**
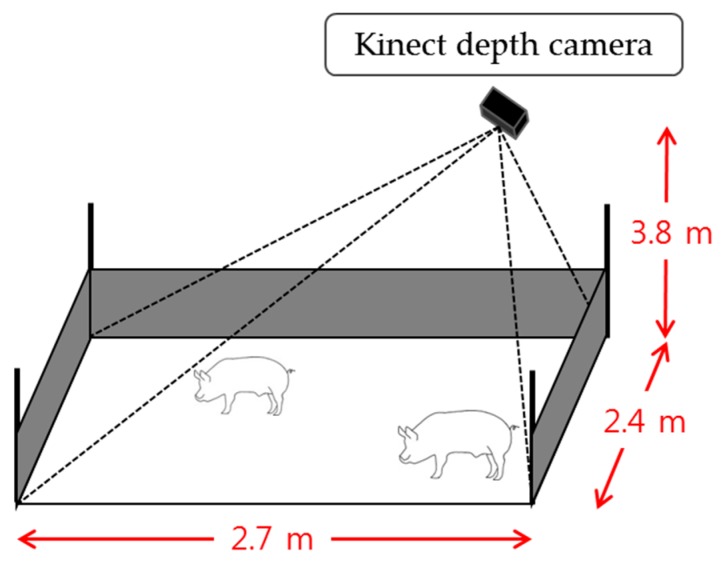
Experimental setup with a Kinect depth camera.

In order to collect the video sequences in a pig room, we first installed a Kinect depth camera (Version 2.0, Microsoft, Redmond, WA, USA) on a ceiling above 3.8 m from the floor in a 2.4 m × 2.7 m pig room located in Sejong city, Korea. Then, we obtained depth video sequences in the pig room in which 13 weaning pigs were raised. Figure 15 shows the entire monitoring environment in the pig room. 

The video sequences had a length of 10 minutes obtained at 10 am (i.e., most pigs show moving activity [60]), which had a resolution of 512 × 424 and 30 frames per second (fps). Although we focused only computation using depth images, we selected daytime (i.e., light-on) images in order to evaluate the accuracy according to the ground truth (derived from the corresponding color images). In addition, a background depth image was obtained to preprocess the foreground segmentation in the video sequences. To reduce the noises, such as holes on the floor, we applied a spatial interpolation technique using a 2 × 2 window, and thus the spatial resolution was reduced to 256 × 212. The details of the background subtraction method can be found in Kim et al*.* [22]. From the detected foreground (i.e., pigs), the moving pigs were extracted using the GMM method. Note that we focus on the pigs’ moving activity to segment the touching-pigs. Among the extracted moving pigs, two adjacent moving pigs were derived through a threshold according to the object’s size (i.e., the number of pixels for the object). In other words, if the object’s size is larger than the threshold of 500, then the object is determined as touching-pigs. Finally, we obtained 3760 touching-pig images, trained YOLO (starting learning rate of 0.1, weight decay of 0.0005, momentum of 0.9, and activation Function of leaky ReLU) with 2260 images, and then tested YOLO and the Image Processing Module with 1500 images.

### 4.2. Results with YOLO Processing Module

**Figure 16:**
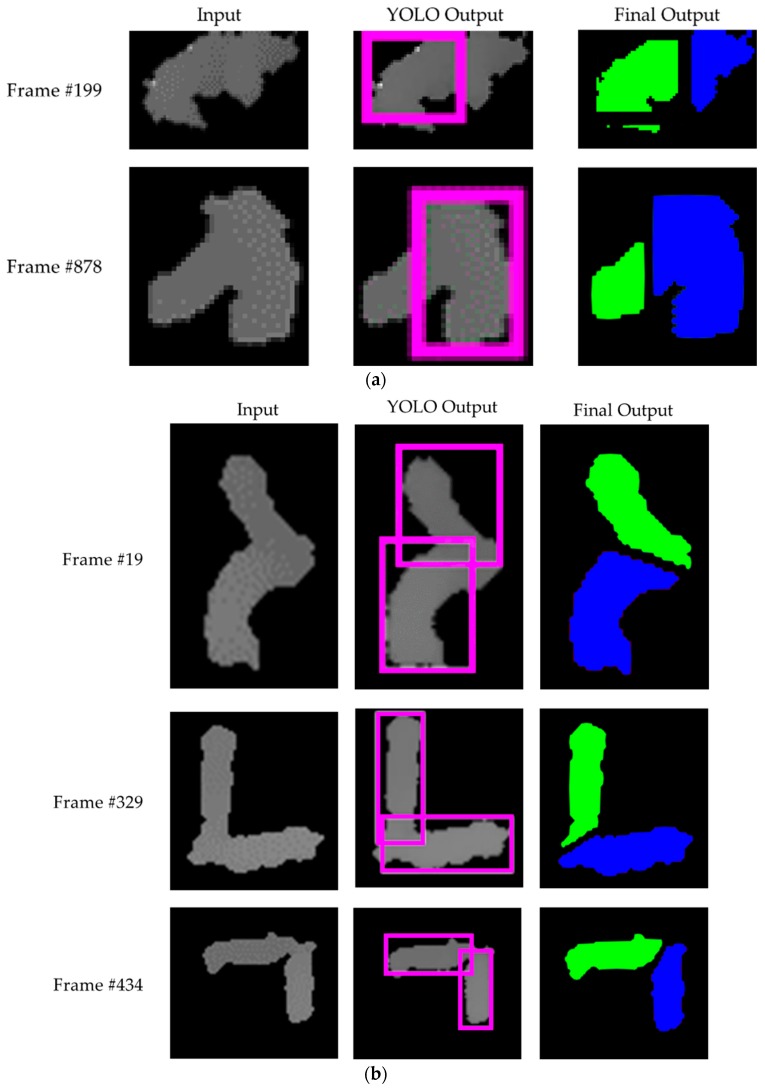
Separation results with YOLO Processing Module: (**a**) separation with a single useful BB; (**b**) separation with two useful BBs.

We first confirmed the separation results (i.e., BB or BBs) of the touching-pigs with test images by only using YOLO9000. Figure 16 shows the segmentation results for touching-pigs using a single useful BB or two useful BBs.

Through the training model with YOLO, a single useful BB or two useful BBs on the touching-pigs were generated in the 1500 test images. Then, touching-pigs were separated using the corresponding segmentation strategies according to the number of BBs. First, in the segmentation strategy of a single useful BB, the touching-pigs were segmented into individual pigs using a boundary line created by projecting the BB into the touching-pigs. Then, the segmentation quality of the segmented pigs was checked by *segmentation_length_th* for a boundary line and *segmentation_size_th* for the segmentation size of the touching-pigs at 20 and 1.5, respectively. We confirmed that the touching-pigs can be separated using a single useful BB in 24 test images, as shown in Figure 16a. 

Second, concerning the segmentation strategy of the two useful BBs, a boundary line created between the two useful BBs segmented the touching-pigs to individual pigs by using Case 1 (i.e., overlapping BBs), because most of the two BBs were produced in an overlapped state on the touching-pigs. After segmenting the touching-pigs by using the boundary line created between the BBs, we evaluated the segmentation quality of the segmented pigs in the same manner of the segmentation strategy of a single useful BB. Finally, we confirmed that the segmentation quality of the segmented touching-pigs was satisfied in 1104 test images, and that the touching-pigs were separated using two useful BBs, as shown in Figure 16b. 

As a result, from 1500 touching-pig images, the proposed method separated 1128 images with the YOLO Processing Module. Although both single useful BB and two useful BBs can separate the touching-pigs, the image processing method was required to improve the accuracy rate. 

### 4.3. Results with Image Processing Module

**Figure 17:**
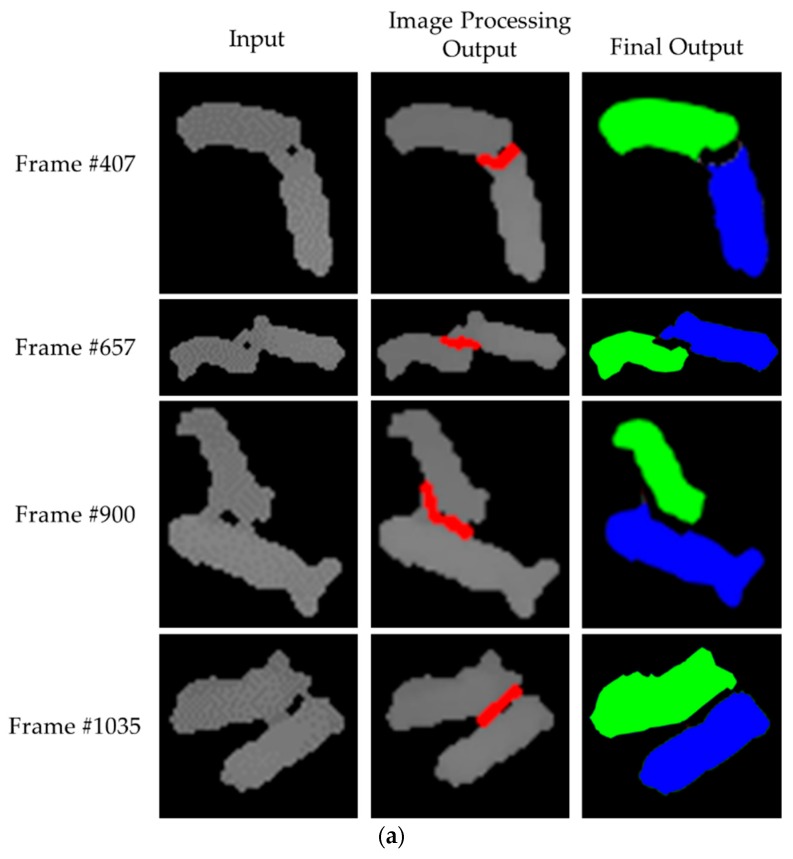

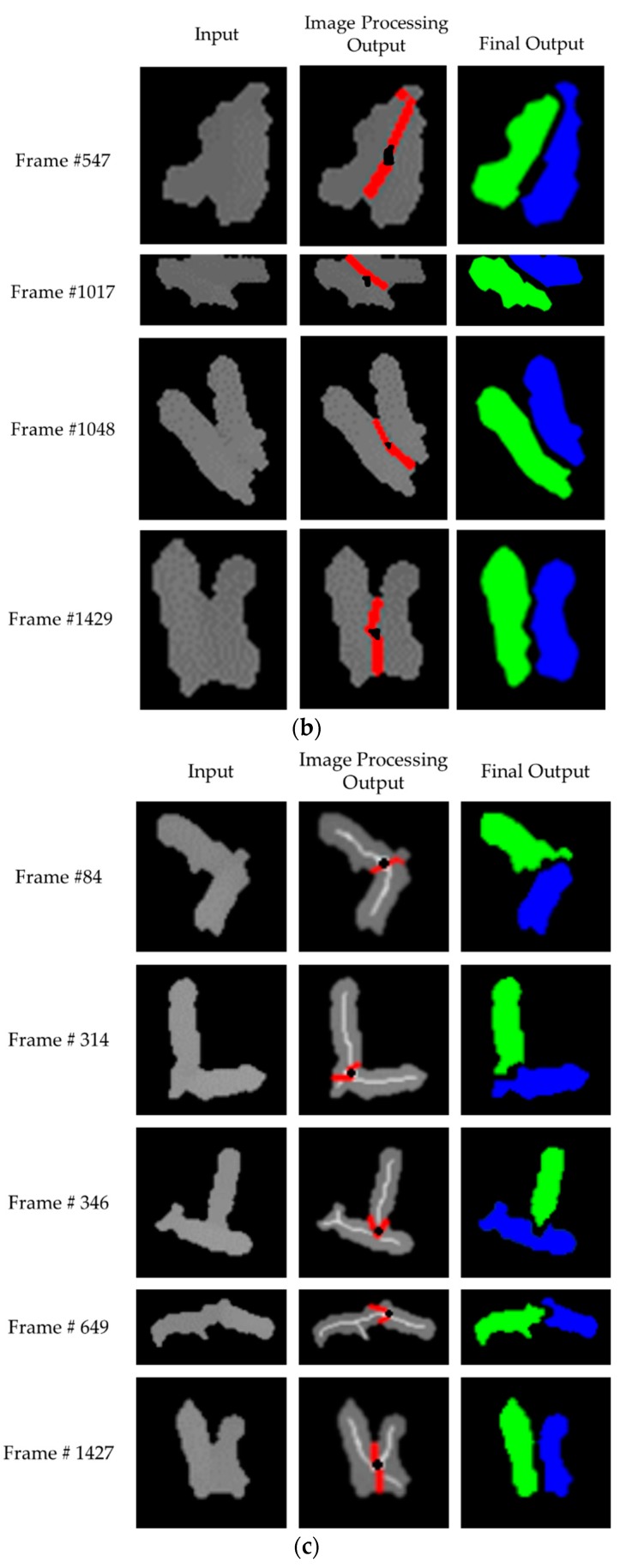
Separation results with the Image Processing Module: (**a**) separation with natural holes; (**b**) separation with artificial holes using the erosion operator; (**c**) separation with artificial holes using the skeleton operator.

Contrary to Section 4.2, we performed only the Image Processing Module without the YOLO Processing Module. In 70 depth images, a natural hole in the touching-pigs was presented according to the touching shape between the pigs. In this case, a guideline could be generated in the natural hole and connected to the CPs or the nearest points, which would be located on the outline of the touching-pigs using a convex hull algorithm. Based on the connected guideline, the touching-pigs were separated as individual pigs. However, in 1430 depth images, we needed to consider a case in which the natural hole did not occur on the touching-pigs. To separate the touching-pigs in the case, artificial holes should be generated using the erosion operator or MAT algorithm. Note that the erosion operator is basically exploited to generate artificial holes, while the MAT algorithm is used when the erosion operator cannot generate the artificial holes. We repeatedly executed the erosion operator eight times to generate artificial holes, and then artificial holes were derived in 282 depth images. In the artificial holes, some guidelines were connected to the CPs or the nearest points of the touching-pigs. Accordingly, the touching-pigs were separated as individual pigs by using the connected guidelines. Meanwhile, in the remaining 1148 depth images, a MAT algorithm was exploited to generate artificial holes, because the holes were removed by the erosion operator through eight repetitions. Using the MAT algorithm, artificial holes could be generated in the region of the touching-pigs, and then the touching-pigs were separated in a manner similar to the above procedures. Figure 17 presents the separation results of the touching-pigs in some sequences by using the Image Processing Module.

### 4.4. Evaluation of Segmentation Performance

**Figure 18:**
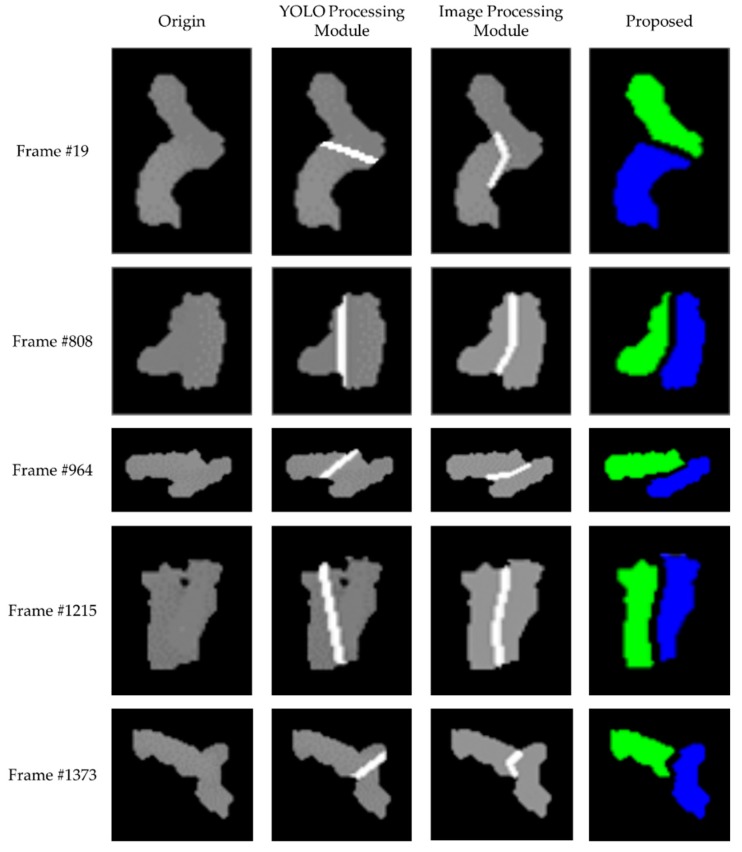
Comparison of the separation results with YOLO Processing Module and the Image Processing Module.

From 1500 touching-pig images, the proposed method separated 1128 images with the YOLO Processing Module and 372 images with the Image Processing Module, based on the BB evaluation and segmentation quality evaluation steps explained in Section 3.1. Figure 18 shows the segmentation results of some cases with the YOLO Processing Module and Image Processing Module. Generally, the YOLO Processing Module produced acceptable results with short boundary line cases (i.e., the touching region between two pigs was relatively small), and unacceptable results with long boundary line cases (i.e., the touching region between two pigs was relatively large in frame #808 and #1215). However, the actual segmentation results depended on the quality of each BB generated from the YOLO detector. For example, both the YOLO and Image Processing Modules could produce acceptable results with a short boundary line case (Frame #964). For some of the short boundary line cases, the YOLO Processing Module could produce a better result (Frame #19), while in others, the Image Processing Module could produce a better result (Frame #1373). Another aspect of the YOLO Processing Module is that it can provide much faster results (by a factor of 200) than the Image Processing Module. In the proposed method, we carefully evaluated the quality of each BB generated from the YOLO detector such that the hybrid solution (i.e., check-YOLO-first-then-Image-Processing) can provide superior performance in terms of both accuracy and execution time. 

**Figure 19:**
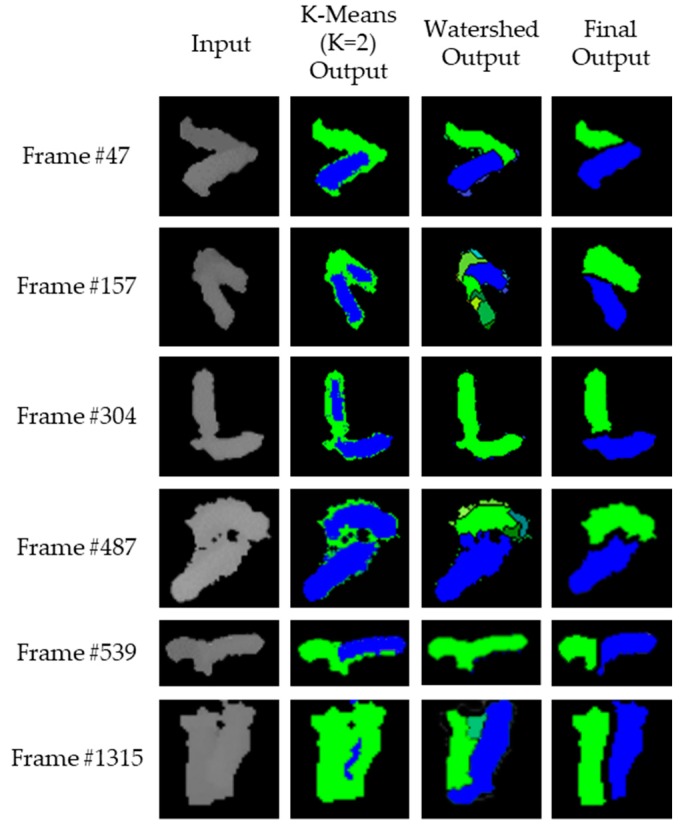
Comparison of separation results with typical methods and the proposed method.

We compared the proposed method with the k-means method [36,46,51] and watershed method [34,35,37,41,56,58] implemented in OpenCV, which is often used to separate touching objects for qualitative analysis. The segmentation results of some typical cases are shown in Figure 19. Although the typical methods were fast enough for real-time performance, it was difficult to assign an individual ID to the result of the segmentation for some cases. As shown in Figure 19, for example, the k-means method could assign each ID to two pigs (i.e., one pig based on a blue color and another pig based on a green color) for Frame #47 and Frame #539, whereas we could not assign an individual ID for Frame #157 and Frame #304. It is also commonly known that the watershed method has an over-segmentation problem. However, as shown in Figure 19, it had over-segmentation (e.g., Frame #157) and under-segmentation (e.g., Frame #304 and #539) problems in cases with touching-pigs. In fact, the depth difference within touching-pigs was not observed by a watershed method in the under-segmentation cases. Although a few boundary pixels were assigned to the blue color for Frame #304 and Frame #539, such few blue pixels could not be used to make an acceptable ID assignment. We confirm that the proposed method can provide a superior performance compared with the typical methods, regardless of various touching-pigs cases.

To produce the quantitative analysis, we also evaluated the performance of each method. The problem of the touching-pigs separation can be interpreted as a problem of ID assignment for each pixel of the touching-pigs. Therefore, we formally define *accuracy* as the ratio of the number of pixels having the correct ID (based on the ground truth) to the size of the touching-pigs, instead of typical precision/recall metrics that are used for object detection problem. Table 2 shows the accuracy and execution time of each method with 1500 touching-pig images. To evaluate the effectiveness of the proposed method, we measured the performance of the YOLO Processing Module and the Image Processing Module separately. That is, the *YOLO Processing-only* method shown in Table 2 means the performance of the YOLO Processing Module with all 1500 touching-pigs images, whereas the *Image Processing-only* method shown in Table 2 refers to the performance of the Image Processing Module with all 1500 touching-pig images. Although the YOLO Processing-only method could provide the fastest solution, its accuracy depended on the quality of each BB generated from the YOLO detector. By selecting the better module for a given case carefully at the run time, the proposed hybrid method (YOLO + Image Processing, as shown in Table 2) could provide the best accuracy with the second best execution time. Especially, for the final goal of 24-h continuous monitoring, ID switch is the most critical factor. For all 1500 touching-pigs, the proposed method provided a pixel-level accuracy greater than 85%. Since the pixel-level accuracy greater than 85% did not cause an ID switch, the proposed method could separate all 1500 touching-pigs without any ID switch. Furthermore, the high-speed execution of the segmentation can have a better chance in producing a complete vision system for higher-level analysis in real-time.

### 4.5. Discussion

The proposed method focused on separation of the touching-pigs in real-time using a low-cost camera (i.e., Kinect) in a pig pen. However, there are some issues to be discussed. First, the depth values obtained from Kinect needed to be improved by removing some noises for detecting the foreground (i.e., pigs in the pen). For example, the Kinect camera has limitations in relation to distance and field-of-view, which degrade the depth data obtained from the camera according to the installed location. In the case of the pig pen, the Kinect camera was installed at a height of 3.8 m above the floor to include the whole area of the pen (i.e., 2.4 m × 2.7 m). From the Kinect camera, either noises were generated at some locations (e.g., undefined depth values, as shown in Figure 2a), or the depth values obtained from a location under the same condition were not consistent (e.g., for the same location, different depth values of 76, 112, and 96 were obtained as time progressed). Furthermore, these problems need to be solved in real-time (i.e., we cannot apply time-consuming methods for improving the depth values). To improve the depth values in real-time, we applied a spatial interpolation technique using a 2 × 2 window, and thus, the spatial resolution was reduced to 256 × 212. Then, the spatial interpolation technique was also applied from a current image to two consecutive images (i.e., temporal interpolation), so that it was able to solve the inconsistency problem of the depth values at the same location. The details of the technique can be found in Kim et al*.* [22]. 

Second, we had to consider the low-contrast images obtained from the Kinect camera. As shown in Figure 2a,b, the contrast of the depth image is poorer than that of the gray image converted from the corresponding color image. In fact, any separation method for touching-pigs may produce good performance with the high-contrast images. However, low-contrast images are inevitable with our experimental environment. Furthermore, one of the most widely used techniques (i.e., CLAHE [29]) cannot improve the low-contrast problem (see Figure 2c), even with the heavy computational workload. Therefore, it is very challenging to separate 8-kg weaning pigs in real-time using a Kinect camera installed 3.8 m above the floor. In this study, to solve this problem, we analyze the quality of the YOLO result (i.e., detailed discussion in Section 3.1 and Section 4.2), not the quality of the input image. Thus, we exploited the advantages of both YOLO and Image Processing Modules in order to solve the low-contrast problem in real time. 

Finally, we trained a detection model of pigs using the depth images through YOLO from the scratch (for explanation, we denote this solution as YOLO_scratch_). However, in order to evaluate the effect of the transfer learning, we additionally conducted an initial experiment using the pre-trained model with ImageNet [62] as the parameter transfer [63]. That is, we separated the touching-pigs with the YOLO Processing Module through the pre-trained model with ImageNet (for explanation, we denote this solution as YOLO_transfer_). Based on the additional experiment, we confirmed that YOLO_transfer_ could provide the slightly better performance than YOLO_scratch_ (i.e., 77.50% with YOLO_transfer_ vs. 75.02% with YOLO_scratch_). Note again that the focus of this study is to develop a hybrid method exploiting the advantages of both CNN and image processing techniques in order to separate the touching-pigs in real time. That is, the goal of this study is not to develop a new CNN technique for segmentation (especially for separating touching objects), but rather to develop a hybrid method for segmentation and evaluate its effectiveness in a pig farm environment with the well-known CNN-based object detection technique (i.e., YOLO), by analyzing its result with respect to segmentation and designing a new image processing-based post-processing technique. However, there are many research issues related to transfer learning, and thus improving the accuracy of the YOLO Processing Module with transfer learning will be interesting in future work. Although the theoretical analysis of CNNs is not within the scope of this study, CNNs are computing progressively more powerful invariants as depth increases [64], and this needs to be considered in future work that develops new CNN techniques. For example, we conducted an experiment by varying the number of training iterations with YOLO_scratch_. The accuracy varied with the number of training iterations (i.e., 73.90% with 10,000 iterations, 75.02% with 50,000 iterations, 74.05% with 100,000 iterations, 70.03% with 200,000 iterations, and 68.18% with 300,000 iterations), while the memory requirement was independent of the number of training iterations. That is, we could empirically derive the best number of training iterations. Although theoretically analyzing the invariant property of YOLO is out of scope of this study, the solid theory of CNNs is still lacking [65], and thus will make for an interesting future work.

## 5. Conclusions 

The touching-pigs segmentation in a surveillance camera environment is an important issue to efficiently manage pig farms. However, with a straightforward method, touching-pigs cannot be separated accurately in real-time due to the relatively low-contrast images obtained from a Kinect depth sensor. 

In this study, we focused on separating touching-pigs in real-time using low-contrast images in order to analyze individual pigs, with an ultimate goal of achieving 24-h continuous monitoring. That is, we proposed a method to separate touching-pigs without the need for time-consuming techniques. In the YOLO Processing Module, the quality of each bounding box generated from the YOLO detector was evaluated and selected. Then, the selected bounding boxes were used to separate the touching-pigs individually according to their relative positions in the bounding boxes. If the results of the YOLO Processing Module were suspect, then we detected a possible boundary line between the touching-pigs in the Image Processing Module. In particular, we utilized the hole and concavity information to find the possible boundary line in “difficult-to-separate” cases. In other words, the proposed method can improve the performance of the separating technique by applying both the YOLO method and the image processing method as a hybrid approach, rather than using only the YOLO method or using only the image processing method.

Based on the experimental results for 1500 touching-pigs obtained over 10 daytime minutes, we confirmed that the separation accuracy was much higher than the typical object segmentation methods (i.e., 67.49% and 49.28% of the k-means method and watershed method, respectively). Furthermore, we also confirmed that the execution time of the proposed method was more than twice as fast as the typical segmentation methods. That is, we could correctly separate all of the touching-pigs without any ID switch (while the ground truth-based accuracy was 91.96%) in real-time. By extending this study, we will develop a separation method for more than three touching-pigs, and finally a real-time 24-h individual pig tracking system for individual pig care. In addition, the proposed method to separate the touching-pigs can be extended to separate other touching objects, such as cells with low-contrast images in real-time.

## Figures and Tables

**Table 1:** Some of the segmentation results (published during 2012–2017). YOLO: You Only Look Once.

Application	Data Type	Data Size	Algorithm	Management of Touching Objects	No. of Objects	Execution Time (Seconds)	Reference
Biomedical	2D (Gray/Color)	256 × 256	Voting	No	Not Specified	540	[31]
		512 × 512	Hierarchical Bayesian	No	Not Specified	300	[32]
		91 × 103	Finite Element Method (FEM)	Yes	3	540	[33]
		Not Specified	Watershed	Yes	117	1	[34]
		1360 × 1024	Watershed	Yes	47	2	[35]
		Not Specified	K-Means/Gradient Vector Flow (GVF)/Snake	Yes	253	125	[36]
		1000 × 1000	Watershed	Yes	104	90	[37]
		Not Specified	Pulse Coupled Neural Network (PCNN)	Yes	554	20	[38]
		1024 × 1280	Saliency Map	Yes	396~610	Not Specified	[39]
		1344 × 1024	Active Contour	Yes	496	Not Specified	[40]
		2080 × 1542	Watershed/Mean Shift	Yes	Not Specified	Not Specified	[41]
	3D (Gray/Color)	408 × 308 × 308	Deformation	No	Not Specified	330	[42]
		256 × 256 × 171	Active Contour	No	Not Specified	300	[43]
		Not Specified	Conditional Random Field (CRF)	No	Not Specified	300	[44]
		Not Specified	Level-Set	No	Not Specified	Not Specified	[45]
		167 × 172 × 39	K-Means	Yes	610	37	[46]
		250 × 250×250	Delayed Agglomeration (DA)	Yes	Not Specified	162	[47]
		1024 × 1256 × 91	Maximum Intensity Projection (MIP)	Yes	Not Specified	180	[48]
		1824 × 834 × 809	Region Competition	Yes	Not Specified	60 (128 Central Processing Units (CPUs))	[49]
Non-Bio/Medical	2D (Gray/Color)	2000 × 2500	Active Contour	No	Not Specified	18	[50]
		800 × 600	K-Means/Level-Set	No	Not Specified	2	[51]
		Not Specified	Butterworth Filter	No	Not Specified	Not Specified	[52]
		Not Specified	Deep Convolutional Neural Network (DCNN)	No	Not Specified	Not Specified	[53]
		640 × 480	Active Contour	No	1	79	[54]
		Not Specified	Active Contour/Graph Cut	No	1	Not Specified	[55]
		Not Specified	Watershed	No	Not Specified	5	[56]
		Not Specified	Linear System	Yes	12	39	[57]
		1000 × 1000	Watershed	Yes	Not Specified	Not Specified	[58]
	2D (Depth)	640 × 480	Active Contour	Yes	12	2	[59]
		512 × 424	YOLO/Shape	Yes	13	**0.001**	Proposed Method

**Table 2:** Comparison of accuracy and execution time.

Method		Accuracy (Avg)	Execution Time (Avg)
K-Means		67.49%	15.38 ms
Watershed		49.28%	2.22 ms
Proposed	YOLO Processing-only	75.02%	0.75 ms
	Image Processing-only	88.78%	4.50 ms
	YOLO+Image Processing	91.96%	1.13 ms
